# Modulating immune response for the prevention and treatment of type 1 diabetes

**DOI:** 10.3389/fimmu.2026.1715863

**Published:** 2026-02-16

**Authors:** Dilrasbonu Vohidova, Prasi Desai, Alvaro Moreno Lozano, Omid Veiseh

**Affiliations:** 1 Department of Bioengineering, Rice University, Houston, TX, United States; 2 Department of Chemical and Biomolecular Engineering, Rice University, Houston, TX, United States; 3 Rice Biotechnology Launch Pad, Rice University, Houston, TX, United States

**Keywords:** immune modulation, immunotherapies, islet transplantation, prevention of type 1 diabetes, type 1 diabetes

## Abstract

In type 1 diabetes (T1D), chronic autoimmune responses lead to the destruction of β-cells in pancreatic islets. As more of the β-cell mass is destroyed, the disease progresses, resulting in insulin deficiency. Recent discoveries uncovering the mechanisms behind the autoimmune attack on β-cells have allowed for a better understanding of the development of the disease and categorizing it into stages of progression. Further, FDA approval of the first drug for the prevention of T1D demonstrated the potential for early intervention therapies. Meanwhile, for patients whose β-cell mass is fully destroyed, islet transplantation has been shown to achieve long-term insulin independence. However, this procedure requires lifelong immunosuppression, which can increase the risk of infections and malignancies. Here, we will review recent advances in immunomodulation approaches for the prevention of type 1 diabetes and strategies for islet cell replacement. First, we introduce the pathogenesis of T1D and the stages of the disease that require different immunomodulatory approaches. Then, we will discuss current immunotherapies for the prevention of T1D, highlighting strategies such as antigen-specific, immune blockade, and cell-based therapies, which aim to stop autoimmune attack for patients at early stages of T1D. Afterwards, we discuss advancement for islet replacement approaches, highlighting islet engineering, device encapsulation, and immunomodulatory biomaterials, which aim to prolong the survival of transplanted islets and minimize the need for immunosuppression. We expect this review to provide a comprehensive understanding of current advances in immunomodulatory therapies for T1D.

## 1 Introduction

Type 1 diabetes (T1D) is a chronic autoimmune disease in which insulin-producing islet β-cells are destroyed by the host immune system, causing the lifelong need for exogenous insulin supply. T1D affects more than 9.5 million people worldwide, and that number is expected to grow to between 13.5 and 17.4 million individuals by 2040 (1, 2). Additionally, the incidence of T1D is increasing in both youth and older adults (3, 4). For instance, T1D incidence among youth aged 10–24 years has grown from 7.78 in 1990 to 11.07 in 2019 per 100,000 individuals (3). At the same time, several studies have demonstrated a peak in T1D incidence among older adults (4). Of the 503,000 newly diagnosed cases of T1D in 2024, 56% were adults, further emphasizing the increasing incidence of T1D in that population (5).

T1D development tends to occur in individuals with a genetic predisposition triggered by environmental or immunological events. Genetic predisposition is determined by specific combinations of genes in the human leukocyte antigen (HLA) region, which affect how T cells recognize and tolerate foreign and self-molecules (6). It is also influenced by polymorphism in genes encoding protein tyrosine phosphatase (*PTPN22*) (7), cytotoxic T lymphocyte-associated antigen 4 (*CTLA4*) (8, 9), IL-2 receptor subunit a (*IL2Ra*) (10) and other genes involved in regulating immune responses. Additionally, polymorphism in insulin (*INS*) leads to the escape of autoreactive T cells from the thymus (11, 12). These genetic factors play a crucial role in shaping the immune response.

**Figure 1:**
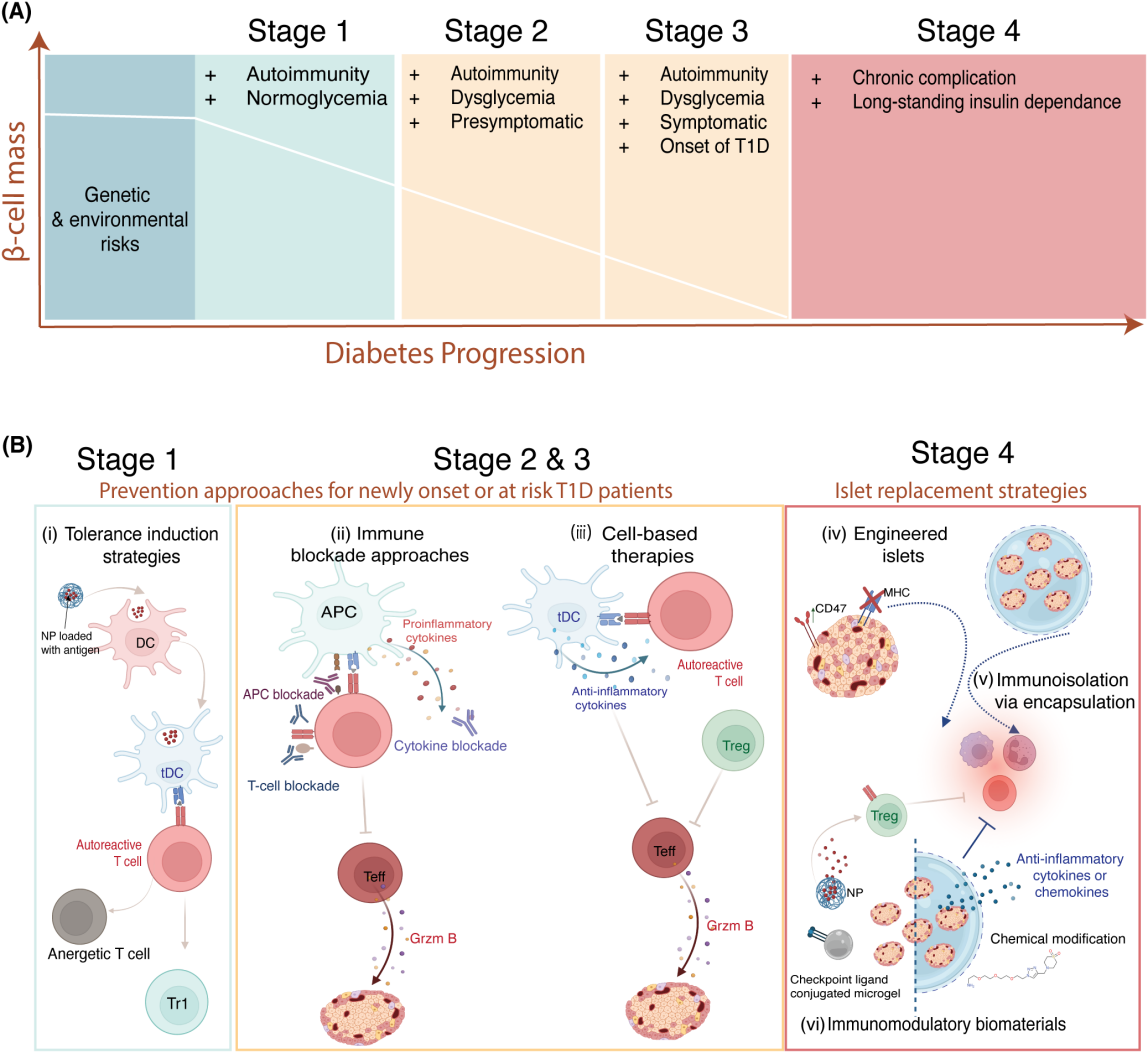
The immunomodulatory strategies as Type 1 diabetes progresses. **(A)** Type 1 diabetes progresses as the β-cells mass is lost. There are several stages of T1D: Stage 1 is characterized by two or more autoantibodies; Stage 2 includes two or more autoantibodies along with β-cell dysfunction: Stage 3 corresponds to clinical T1D; and Stage 4, which is long-standing T1D. **(B)** Approaches for the prevention of the onset and β-cell replacement as the disease progresses. (i) Antigen-specific strategies utilizing autoantigens have been investigated for individuals at stage 1 or at risk of T1D. These approaches present antigens in a non-inflammatory environment to shift the phenotype of autoreactive T cells by anergy or tolerizing them to become type 1 regulatory cells. These approaches rely on tolerogenic dendritic cells. Hence, nanoparticles that can shift dendritic cell phenotype to become tolerogenic are often utilized for antigen delivery. (ii) While for stage 2 & 3 T1D patients, immune blockade approaches targeting T cells, antigen-presenting cells, and proinflammatory cytokines could be promising to prevent autoimmune attack on β-cells. (iii) Additionally, cell-based therapies utilizing regulatory T cells and tolerogenic dendritic cells have the potential to inhibit the activity of autoreactive effector T cells in stages 2 & 3 of the disease. (iv-vi) For patients at the later stage of T1D, β-cell replacement utilizing engineered islets, encapsulation in biomaterials, and utilizing immunomodulatory biomaterials to enhance islet graft survival have been explored. (iv) Islets have been engineered to downregulate MHC class and overexpress CD47 to prevent their recognition and destruction by the immune system. (v) Alternatively, encapsulation of islets can mitigate immune clearance, which has been investigated in the clinic. (vi) Biomaterials incorporating chemically modified coating, immunomodulatory agents (including cytokines, chemokines, checkpoint ligands), and immunomodulatory drugs have been explored for long-term graft protection. NP, nanoparticle; tDC, tolerogenic dendritic cells; Tr1, type 1 regulatory T cell; Grzm B, granzyme B; Teff, effector T cell; Treg, regulatory T cell; MHC, major histocompatibility complex.

T1D development is closely linked with β-cells mass destruction, and the disease progresses as more β-cells are lost (**Figure 1A**) (13, 14). For earlier stages of T1D, where most of the β-cells’ mass is still intact, there is an opportunity window for immunotherapies to prevent further autoimmune attack and destruction of β-cells (15). For advanced T1D patients, where β-cell mass is severely reduced, maintaining normoglycemia with insulin is challenging (16). The recent FDA approval of Lantidra, the first allogeneic islet therapy for T1D, established islet transplantation as a viable option for some patients, enabling tighter blood glucose control (17). This was a significant milestone for the field as it was the first cell therapy approved for the treatment of T1D. However, this procedure requires extensive immunosuppression to prevent islet graft rejection. The heavy immunosuppressive regimen puts the patient at risk of infections, malignancies, worsening islet graft function, and organ damage (18–20). This highlights the urgent need for novel interventions to prevent autoimmune attack on β-cells and delay disease progression. In parallel, innovative strategies must be developed to support the long-term survival of transplanted islets in advanced-stage patients without systemic immunosuppression.

This review aims to provide a comprehensive overview of immunomodulatory approaches across all stages of T1D progression. First, we will discuss prevention strategies, such as antigen-specific, immune blockade, and cell-based therapies, which aim to protect endogenous β-cells. Then we will review the strategies for islet replacement, which are necessary at the later stages when majority of β-cells mass is destroyed, including utilizing engineered islets, islet encapsulation in macro or micro device and utilizing immunomodulatory biomaterials for long-term survival graft islets (**Figure 1B**). These approaches are inherently linked, as advances in immune modulation directly impact the success and durability of β-cell replacement therapies by preventing graft rejection and recurrent autoimmunity. Overall, we expect this review to provide an extensive summary of the latest advancements and remaining gaps in the field of immunomodulation for prevention and cure for T1D.

## 2 Immunological mechanism of T1D

Although the immune mechanisms leading to β-cell autoimmunity are heterogeneous, T1D is believed to be a T-cell-mediated autoimmune disease. The defects in both central and peripheral tolerance lead to the generation of autoreactive T cells (12, 21). In central tolerance, naïve T cells travel from the bone marrow to the thymus, undergoing positive and negative selection to develop into CD8 or CD4 cells. T cells acquire their T-cell receptor (TCR) after the positive selection stage, where they demonstrate that they recognize MHC molecules. This is followed by negative selection, where a broad array of peripheral tissue self-antigens is displayed by medullary thymic epithelial cells (mTECs) to deplete self-reactive T cells from the pool, which demonstrate high-affinity TCR binding to MHC presenting self-antigen (22). In the case of T1D, various parameters can influence the efficiency of negative selection. For instance, low expression of insulin due to the polymorphism at the *INS* gene promoter results in defective negative selection of insulin-reactive T cells. At the same time, expression of the *PTPN22* gene leads to diminished TCR signaling and consequent reduction of apoptosis of autoreactive T cells (23, 24).

**Figure 2:**
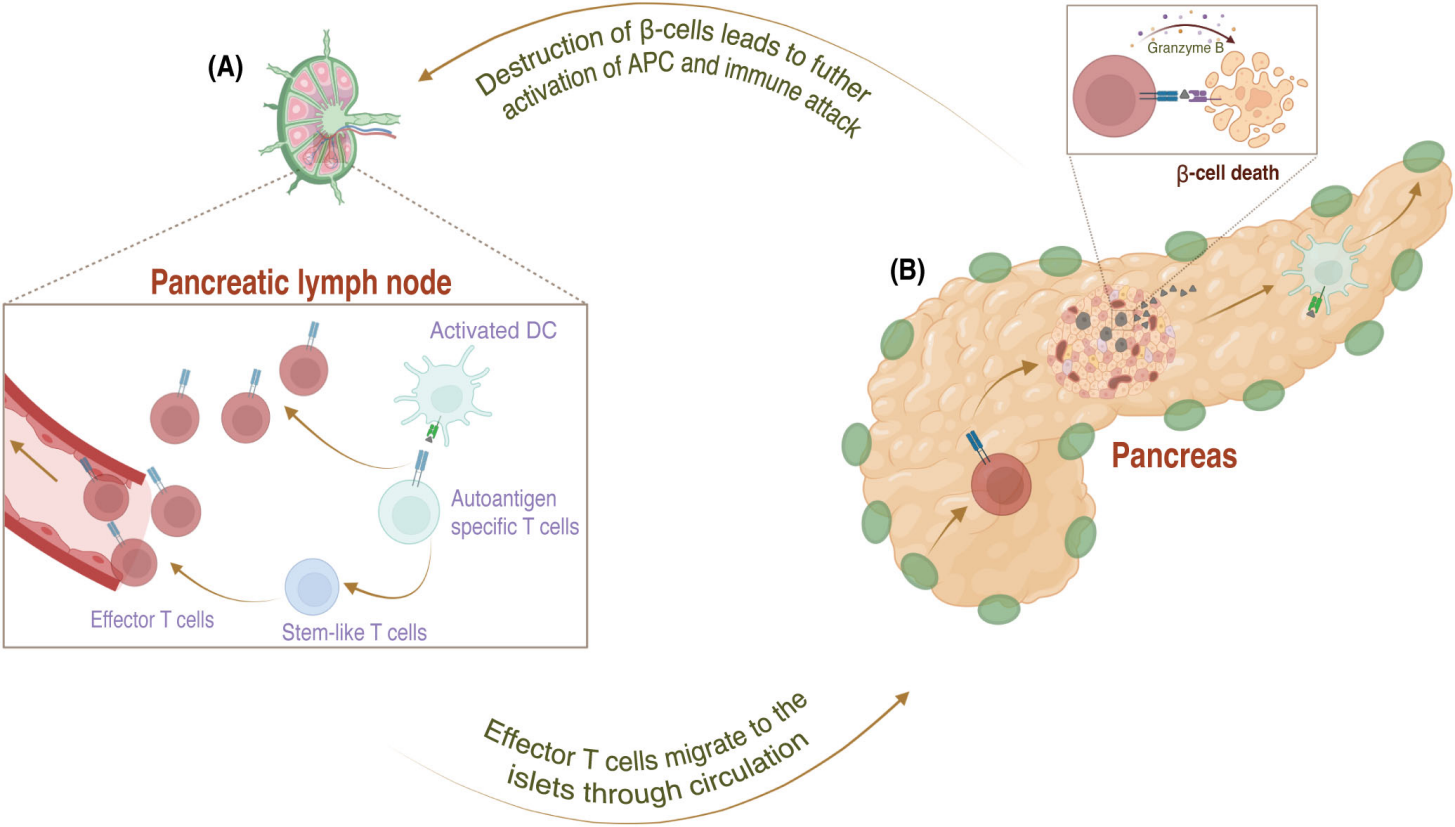
The mechanism of pathogenesis in Type 1 diabetes in the periphery. **(A)** In the pancreatic lymph node, APCs activate these autoreactive T cells, which escape negative selection, by presenting antigens from damaged β-cells and providing a co-stimulatory signal via secretion of proinflammatory cytokines. Activated autoreactive T cells differentiate into effector cells and migrate to the pancreatic islets through the bloodstream. **(B)** In the pancreas, effector T cells attack the β-cells by secreting interferon (IFN)-γ and granzyme **(B)** The initial destruction of β-cells leads to a vicious cycle that fuels the immune attack and speeds up the destruction of even more β-cells.

Once autoreactive T cells reach the periphery, dendritic cells in the pancreatic draining lymph nodes serve as antigen-presenting cells (APCs) and present fragments of islet molecules from damaged β-cells to these T cells. Additionally, these dendritic cells secrete cytokines interleukin (IL)-12 and -15, enhancing the expression of costimulatory molecules and activating the autoreactive T cells (11, 25). Activated autoreactive T cells then differentiate into effector T cells and migrate to the islets through the circulation. A small population of autoreactive T cells exists in the pancreatic lymph node as stem-like T cells continuously sustaining anti-islet immunity. This is important because effector T cells have a short lifespan and do not persist once they reach the islet niche (12, 25, 26). Upon reaching the islets, the autoreactive effector T cells initiate insulitis, an infiltration of immune cells into the pancreatic islets, marking immune-mediated β-cells for destruction. During this process, autoreactive effector CD8 cells destroy β-cells by secreting IFNγ and granzyme B (**Figure 2**), while CD4 cells contribute by secreting proinflammatory cytokines (21, 26–30). Although immune cells play an essential role in the destruction of β-cells, recent findings also suggest that β-cells themselves are prone to self-destruction due to their susceptibility to stress and limited self-defense (21). This suggests that escaped autoreactive T cells, in tandem with susceptible β-cells, may lead to autoimmunity in T1D.

## 3 Prevention approaches for T1D

Recent advancements in early genetic and antibody-based screening, coupled with an enhanced understanding of the pathogenesis of disease, have significantly accelerated the progression in T1D prevention efforts (31, 32). Consequently, the ability to identify at-risk patients allowed for the development and evaluation of stage-specific prevention strategies. For instance, antigen-specific strategies have been explored for patients at the early stage of T1D to alter autoreactive T-cell response. This approach relies on utilizing antigens presented in a tolerogenic context to silence autoreactive T cells (33). For individuals at stage 2 and stage 3, approaches such as immune blockade and cell-based immunotherapies have been pursued. The immune blockade approaches encompass monoclonal antibodies that target T cells, antigen-presenting cells, or proinflammatory cytokines, thereby preventing these immune components from driving disease progression. Meanwhile, cell-based therapies involve engineering immune cells, (e.g., Tregs, dendritic cells) immunomodulatory stromal cells (e.g., MSCs) or platelets to enhance their tolerogenic function to prevent autoimmune attack (**Figure 1B**). Together, these approaches reflect a shift toward precision prevention in T1D, emphasizing the importance of tailoring immunotherapeutic interventions to the underlying immunological landscape and disease stage. However, for individuals in stage 4, the research focus shifts towards islet replacement strategies. A major barrier in this field has been the reliance on systemic immunosuppression, which carries significant risks. To address this, next-generation therapies have been developed to achieve localized immunomodulation.

### 3.1 Antigen-specific

As preclinical models have allowed a greater understanding of T1D disease mechanisms, antigen-specific strategies have become a point of interest. By presenting the autoantigens in a tolerogenic environment through controlled delivery of the antigens themselves or manipulation of immune cells, antigen-specific aims to combat autoimmune responses. Many antigen-specific strategies are discussed in this review, from antigen-specific regulatory T cells to tolerogenic dendritic cells pulsed with personalized peptides. This section focuses on autoantigens, the most notable being insulin, which has seen much exploration in clinical trials (34).

Autoantigens, or self-antigens, are produced within the body and are recognized as threats to the immune systems of patients with autoimmune diseases. The most prominent autoantigens used to test for T1D, which are now targeted in clinical trials for T1D prevention, are insulin, proinsulin, and glutamic acid decarboxylase (GAD65) (**Table 1****) (**35). In recent years, efforts have mainly been centered on assessing whether insulin administration can affect immune tolerance in high-risk, younger patients as a preventative measure for T1D development. Studies in NOD mice revealed that insulin can achieve tolerance by elevating anti-inflammatory cytokine levels and Tregs (77). NOD mice, widely regarded as the gold standard preclinical model for Type 1 Diabetes, exhibit disease pathogenesis that closely mirrors human T1D, making them invaluable for studying autoimmunity and evaluating therapeutic strategies (78, 79). In 2015, Bonafacio et al. demonstrated that high oral insulin doses in children were safe, causing no hypoglycemia, and induced a protective immune profile characterized by an elevated ratio of regulatory T cells to IFN- γ-expressing cells (80). Since then, studies have found that oral insulin is safe in both children and adults, with the most recent clinical study finding oral insulin to reduce blood glucose levels in adults (36, 41). However, the efficacy of oral insulin as a standalone treatment requires further exploration, as it has shown mixed results across clinical trials. For instance, in the trial involving infants at risk of T1D, oral insulin was ineffective in preventing the development of islet autoantibodies (37). While in another trial, oral insulin was shown to be effective only in a subset of patients with high levels of IA-2 autoantibodies (38). Currently, another clinical trial is investigating a different insulin administration route (intramuscular), utilizing insulin B chain with MAS-1 adjuvant (39).

**Table 1:** Current therapies in clinical trials for the prevention of the onset of diabetes.

Target	Treatment name(s)	Progress	Limitations	Status	References, Clinicaltrials.gov identifier
Insulin	Oral insulin	Safety objective reached	Treatment not associated with immune response	Phase 2 completed 2017 Phase 2 completed 2024	(35–38), NCT02547519 NCT00419562 NCT03364868
Insulin B chain	MER3101	Results not posted	Results not posted	Phase 1 in progress	(39), NCT03624062
Proinsulin	Gold nanoparticles conjugated with proinsulin peptide	Safe and well tolerated	Small number of participants	Phase 1 completed 2019	(40), NCT02837094
GAD65	GAD-alum with Vitamin D/D3 supplementation	Preserves C-peptide levels;	Efficacious in genetically selected group	Phase 2 trials completed 2021	(41), NCT03345004
CD3 on T cells	Teplizumab (anti-CD3 monoclonal antibody)	Delayed T1D progression in stage 2 patients	Most patients experience adverse effects	Teplizumab FDA approved (2022)	(42–44), NCT03875729, NCT01030861
CD2 on T cells; inhibits T cell stimulation with CD58	Alefacept (LFA-3-Ig)	Insulin secretion preserved long-term in 30% of patients	β-cell function not preserved	Phase 2 trial terminated	(45, 46), NCT00965458
T-cell surface markers	Anti-thymocyte globulin (ATG)	Preserves β-cell function; CD4 exhaustion	Prevented T1D in only 50% of high-risk individuals	Phase 2 completed 2018	(47–52), NCT02215200
CD2 on T cells and NK cells	Siplizumab (monoclonal antibody)	Results not posted	Results not posted	Phase 2 terminated	(53, 54), NCT05574335, NCT06025110
B7; inhibit co-stimulation of T cells through interaction of B7 with CD28	Abatacept (CTLA4-Ig)	Preserved insulin secretion and reduced follicular helper T cell population	Did not delay T1D progression, adverse effects	Phase 2 completed 2022	(55–59), NCT00505375, NCT01773707
CD40 on antigen presenting cells	Frexalimab (anti-CD40L)	Results not posted	Results not posted	Phase 2 trial recruiting	(60) NCT06111586
CD20 on B cells	Autologous Tregs + Rituximab (anti-CD20 monoclonal antibody)	Delayed C-peptide level reduction	Adverse effects, did not delay T1D progression	Phase 1/2 trial completed 2022	(61, 62), NCT00279305, NCT06688331
TNF- α	Golimumab (anti-TNF-α monoclonal antibody)	Long-term benefits, lower reduction in C-peptide	Small number of eligible patients	Phase 2 trials completed 2021	(63, 64), NCT02846545, NCT02846545
TNF- α	Etanercept (TNF-α inhibitor), GAD-alum, Vitamin D	Feasible and well-tolerated	No effect on β-cell function	Phase 2 completed 2019	(65), NCT02464033
IL-6	Tocilizumab (IL-6 receptor inhibitor)	reduced T cell IL-6R signaling	Did not slow β-cell loss of function	Phase 2 completed 2020	(66), NCT02293837
IL-8	Ladarixin (IL-8 receptor inhibitor)	Some differences in HbA1C levels	No effect on β-cell function, no change in C-peptide levels	Phase 2 completed 2019	(67), NCT02814838
GLP-1R	Liraglutide + Tregs	Results not posted	Results not posted	Results not posted	(68), NCT03011021
IL-21, GLP-1R	NN-8828 (anti-IL-21), liraglutide (GLP-1 RA)	Changes in C-peptide and HbA1C levels	Therapeutic effects did not last after treatment stopped	Phase 2 completed 2019	(69), NCT02443155
IL-12 and IL-23	Ustekinumab (a monoclonal antibody targeting the p40 subunit)	Well tolerated; higher C-peptide levels	Delayed efficacy	Phase 2 completed 2021; Phase 2/3 in progress	(70) ISRCTN 14274380, NCT03941132
Tregs	Autologous polyclonal Tregs + low dose IL-2	Expands exogenously administered Tregs	Off-target effects	Phase 1 completed 2021	(71), NCT02772679
Tregs	Polyclonal expanded Tregs	Safe	Did not preserve C-peptide levels	Phase 2 completed 2020	(72), NCT02691247
CD4 T cells	PIpepTolDC (Tolerogenic DCs pulsed with islet antigen)	Long-term reduction in T-cell autoreactivity	Assessment pending on if β-cell function is preserved	Phase 1 in progress	(73, 74), NCT04590872
CD8 T cells	AVT001 (Autologous Dendritic Cells)	Less decline from normal C-peptide levels	No significant change in HbA1c and insulin dose	Phase 1/2 completed in 2023	(75), NCT03895996
Inflammatory immune response	Mesenchymal Stem Cells	Increased Treg population, more anti-inflammatory cytokines, improved HbA1C levels	Limited patients meet eligibility criteria	Status unknown, but likely that Phase 1/2 trial completed in 2020	(76), NCT04078308

Another molecule of interest is proinsulin, the insulin precursor. Proinsulin has been proven effective in preserving β-cell function, but its positive immunomodulatory effects are not uniform across patients (35, 81, 82). Recently, Tatovic et al. tested the safety of gold nanoparticles conjugated with proinsulin peptide. Using microneedles as a delivery system, this method was safe in patients, which may provide a possibility to further optimize proinsulin in clinical trials, using the anti-inflammatory properties of gold (40). On the preclinical front, proinsulin has been combined with other immunomodulatory therapies like IL-10 or anti-CD3 to enhance efficacy *in vivo*. These efforts aim to address the challenge with current treatment efficacy being dependent on disease duration at the time of administration and continuous oral administration of proinsulin (83).

GAD65 is another autoantigen critical in T1D disease progression and a potential marker of impaired β-cell function (84). GAD combined with aluminum hydroxide, GAD-alum, has been tested in clinical trials. Salami et al. used GAD-alum in healthy children at risk for diabetes, finding that the treatment was associated with lower levels of cytotoxic and autoreactive T cells long-term. However, the trial was terminated early, leading to a small cohort size (85). Ludvigsson et al. combined intra-lymphatic administration of GAD-alum with vitamin D, an immunomodulatory agent, preserving C-peptide levels, a biomarker for insulin production, in patients of a specific haplotype (86). Another well-established autoantigen involved in T1D pathogenesis is insulinoma-associated protein 2 (IA-2). Its presence has been implicated as a good predictor of T1D, but not much work has been done to assess IA-2’s utility for T1D treatment (87). Oral delivery of the intracellular domain of IA-2 using bacterium-like particles in NOD mice prevented T1D onset (88). Zn-T8 has been explored as a diagnostic tool for T1D, but not as a treatment (89, 90).

More recently discovered autoantigens are islet-specific glucose-6-phosphatase catalytic subunit-related protein (IGRP) and chromogranin A (ChGA). IGRP-specific T cells are upregulated in T1D patients (91). Similarly, changes in ChGA concentration can indicate T1D disease progression (92, 93). In NOD mice, a liposome targeting IGRP delayed T1D onset by lessening the activity of autoreactive T cells (94). ChGA-targeted therapies have not been tested in clinical trials, but ChGA deficiency has been shown to reduce T cell autoreactivity and protect mice from T1D (95). In NOD mice, Jamison et al. tested nanoparticles containing an insulin-ChgA hybrid peptide, which prevented T1D by impairing T cells’ ability to produce proinflammatory cytokines; an increase in Tregs and a decrease in effector T cells (Teffs) was observed. Recent work has identified other potential autoantigen targets involved in T1D that may become more prominent in the future, including toll-like receptor 4 (TLR4), Tetraspanin, prolyl-4-hydroxylase β subunit (P4Hb), insulin gene enhancer protein (ISL-1), and GRP78 (35, 83, 96–101).

These tolerance-inducing strategies, using autoantigens, seem the most highly tested therapeutic among those discussed in this review. However, challenges persist in this area. Namely, recent clinical trials have primarily served as safety checkpoints, with limited efficacy for patient outcomes. Doses, combinatorial regimes, and administration routes must be refined to improve effectiveness. Further, due to the vast array of immune patterns in T1D patients, it is plausible that certain autoantigens are effective in specific patient groups, rather than serving as a universal cure, presenting a screening barrier for widespread adoption (34). The delivery route remains critical, with ongoing trials investigating intravenous, intranasal, oral, and intralymphatic administration. Further research is needed to standardize delivery methods and optimize antigen-specific therapies for different patient subgroups (102). In this area, therapies targeting immune cells may provide an alternate route.

### 3.2 Immune blockade

Immune blockade strategies are among the most prominent efforts to prevent autoimmune-mediated β-cell destruction. These approaches target essential T-cell activation, co-stimulation, and inflammatory signaling pathways to preserve β-cell function. This section explores the mechanisms of action currently targeted under investigation in clinical trials and the challenges limiting the wide use of these therapies.

#### 3.2.1 T-cell blockade

**Figure 3:**
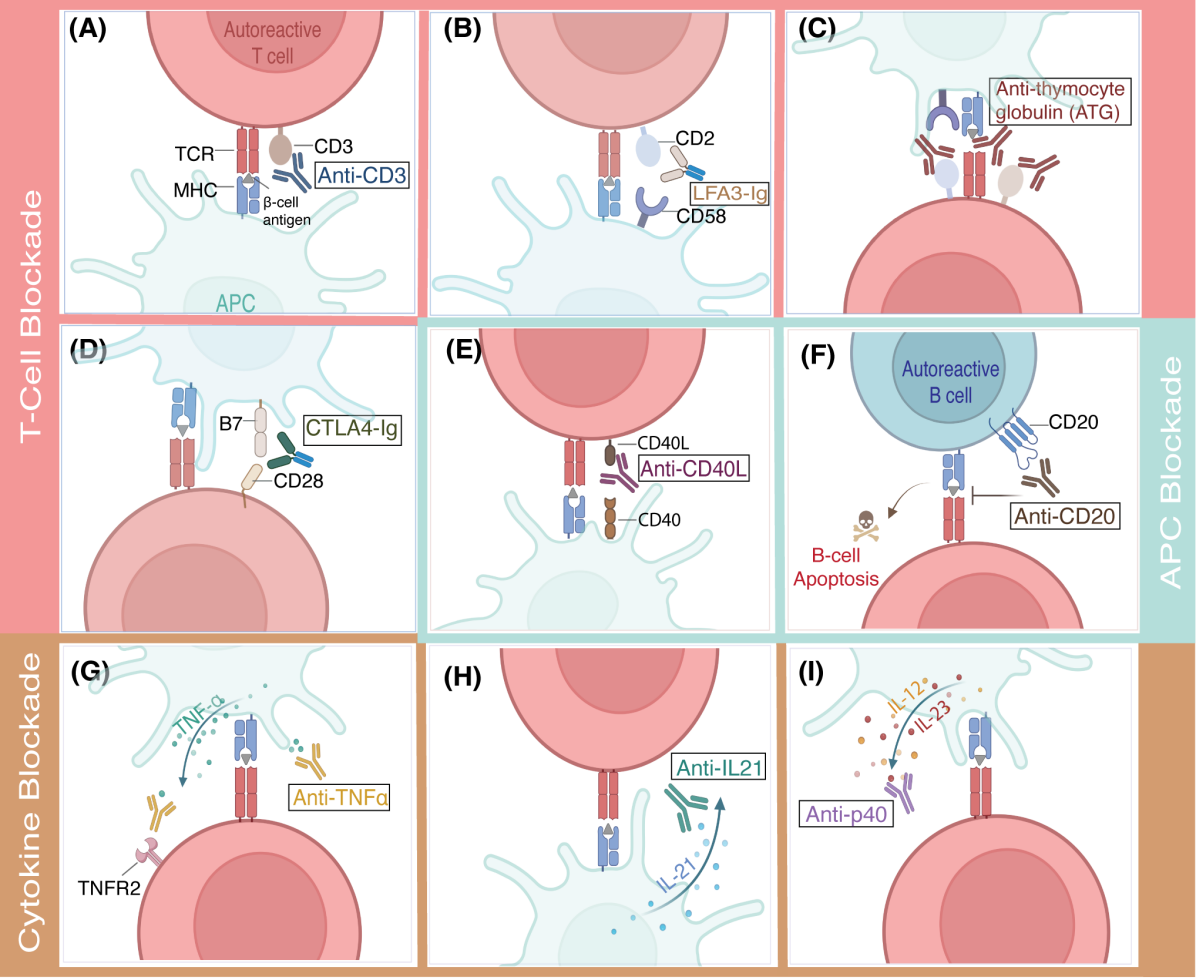
Mechanism of action for promising immunotherapies in clinical trials targeting T cells, APCs, and cytokines for preventing Type 1 Diabetes. **(A-D)** T cell blockade therapies aim to suppress autoreactive T cells. **(A)** Anti-CD3 depletes the T cells by binding to CD3 on T cells and preventing their activation. **(B)** LFA3-Ig antibody prevents T cell activation by targeting CD2 and blocking its interaction with CD58, a critical co-stimulatory signal. **(C)** Anti-thymocyte globulin (ATG) broadly depletes T cells by binding to multiple surface markers, including CD2, CD3, and CD25, etc. **(D)** CTLA4-Ig inhibits T cell activation by binding to B7, preventing its interaction with the co-stimulatory receptor CD28. **(E-F)** APC blockade strategies focus on disrupting the activation of antigen-presenting cells. **(E)** Anti-CD40L blocks the CD40L-CD40 interaction, halting the activation of APCs and subsequent inflammatory signaling. **(F)** Anti-CD20 targets CD20 on B cells, inducing their apoptosis and reducing autoantibody production. **(G-I)** To counteract the pro-inflammatory environment of T1D, cytokine blockade therapies target key inflammatory pathways. **(G)** TNF-α inhibitors behave like TNF receptor 2 or directly block TNF-α, suppressing this pro-inflammatory cytokine’s effects. **(H)** IL-21 inhibitor neutralizes the pro-inflammatory signaling of this cytokine **(I)** while anti-12p40/IL23p40 targets the p40 subunit shared by IL-12 and IL-23.

Several therapies targeting T cells have been investigated to prevent the progression of T1D (**Table 1**). The most notable one is teplizumab, an Fc receptor, non-binding humanized anti-CD3 monoclonal antibody, which received FDA approval in 2022. This was a significant milestone since it was a first drug approved for preventing the progression of T1D in patients at stage 2 of the disease (**Figure 3A**) (42). Treatment with teplizumab comprises a 14-day daily intravenous (IV) course, which has been shown to delay the progression of the disease in 43% of patients for 48.4 months in the phase 2 trial (43). However, due to the nature of the systemic administration of the treatment, multiple adverse events were observed in patients treated with teplizumab. An integrated analysis from five clinical trials revealed that most patients (99.5%) who received the teplizumab course experienced at least one adverse effect associated with the treatment (44). Although adverse events were generally transient, the response to teplizumab among stage 2 patients was heterogeneous (103).

Other therapies targeting the activation of T cells via costimulatory and co-inhibitory signals in new-onset T1D patients, such as LFA-3-Ig, anti-thymocyte globulin (ATG), CTLA4-Ig, have been investigated in clinical trials and showed transient protection of β-cells. For instance, a phase 2 trial in patients with recent-onset T1D treated with LFA-3-Ig fusion protein (alefacept), which binds to CD2 and inhibits T cell co-stimulation with CD58, demonstrated insulin production preserved in 30% of subjects for up to two years post-treatment. LFA-3-Ig was administered via intramuscular injections weekly for two 12-weeks, separated by a 12-week interval. Despite promising results, the trial was terminated due to the discontinuation of production by the drug manufacturer, and enrollment was reduced to 49 participants. (**Figure 3B**) (45, 104). The peripheral blood mononuclear cells (PBMCs) from trial participants revealed that alefacept allowed for the preservation of β-cells by exhausting CD8 T cells. However, the higher numbers or frequencies of islet antigen–reactive CD4+ T cells were associated with poor response to alefacept, suggesting that dosing may be inadequate to eliminate islet antigen-reactive CD4+ T cells in specific individuals (45, 46). While the clinical trials with LFA-3-Ig were terminated, CTLA4-Ig and anti-thymocyte globulin (ATG) are still under investigation (32).

ATG broadly depletes the T cells by recognizing their surface markers, such as TCR/CD3, CD4, CD8, CD25, CD45, HLA class I, and HLA-DR, and binding to them, which leads to T cell killing via complement activation, antibody-dependent cell-mediated cytotoxicity, or activation-induced cell death (**Figure 3C**) (47, 48). Although the FDA has approved ATG to prevent renal allograft rejection (49), its efficacy in delaying the progression of type 1 diabetes is still under investigation. A phase 2 trial by TrialNet demonstrated that a 2-day course of treatment with low-dose ATG-infused IV preserved β-cell function for one year in new-onset stage T1D patients (50). Responders to the treatment revealed CD4 exhaustion and temporarily elevated levels of IL-6, CXCL10, and TNF-α two weeks posttreatment (51). The most recent study with children at stage 2 of T1D treated with a 2-day course of a low dose of ATG demonstrated that three subjects (50%) remained diabetes free after 1.5, 3, and 4 years of follow-up, while the other three developed stage 3 within 1–2 months after therapy (52). Siplizumab, a humanized anti-CD2 monoclonal antibody, has also shown depletion of Teffs and promotion of Tregs *in vitro (*105). Siplizumab was investigated in clinical trials for patients with new-onset T1D; however, the trial was terminated early due to the company’s decision (53). Another dose-finding clinical trial with Siplizumab in patients recently diagnosed with T1D was terminated as well due to excessive lymphodepletion (54).

Humanized CTLA4-Ig fusion protein is a cytotoxic T lymphocyte-associated antigen four fused to modified human immunoglobulin G and is another widely studied treatment for preventing the onset of T1D (106). CTLA-4 binds to B7 molecules on antigen-presenting cells (APCs), thereby inhibiting T cell activation and proliferation by blocking the critical B7-CD28 costimulatory signaling pathway(**Figure 3D**) (107).CTLA4-Ig, abatacept, is already FDA-approved for treating prophylaxis of acute graft versus host disease (108) and rheumatoid arthritis (109); however, similar to ATG, its efficacy for delaying the onset of T1D is still being explored. A recent phase 2 placebo-controlled clinical trial on stage 1 T1D patients who received monthly abatacept IV infusion for 12 months showed that the treatment preserved insulin secretion and reduced the frequency of a subset of ICOS^+^ follicular helper T (Tfh) cells, which previously was reported to be an indication of successful clinical response in T1D patients (55, 56). Inducible co-stimulatory molecule (ICOS) plays a critical role in positively regulating T cell function, and its elevated expression is linked to an increase in circulating peripheral helper T cells (Tph) (57, 58). However, abatacept infusions did not delay the progression of the disease (56). Previously, abatacept has demonstrated promise in stage 3 T1D by delaying the loss of insulin production by 9.6 months when administered over two years. However, its systemic delivery has been associated with infusion-related adverse effects in 22% of patients, highlighting the need for safer and more targeted approaches for immunotherapy (59).

#### 3.2.2 Antigen-presenting cell blockade

Antigen-presenting cells are also targeted to prevent the progression of T1D, as they participate in the development of the disease by priming autoreactive β-cell-pecific T cells and promoting the activation of autoantibody secretors (110–112). Additionally, APCs can lead to islet graft recognition and subsequent rejection (113–115). One of the currently explored APC blockade therapies is the anti-CD40 ligand (CD40L) antibody (**Figure 3E**). CD40L expressed on activated T cells binds to CD40, which is strongly expressed on APCs, such as dendritic cells and B cells. CD40 and CD40L interaction leads to inflammatory responses such as B cell activation and differentiation, production of antibodies and proinflammatory cytokines, and T cell clonal expansion (116–118). Although anti-CD40 therapies have also been extensively investigated, they are less efficacious than anti-CD40L targeting approaches in graft rejection prevention (119). For instance, anti-CD40 has been investigated in clinical trials by Novartis (120). Still, the clinical trial was discontinued at phase 2 because it was less efficacious compared to the established regimen for kidney transplant (121). While CD40L targeting therapies have shown promising results in clinical trials for the treatment of autoimmune diseases such as Sjögren’s disease (122), systemic lupus erythematosus (123), and multiple sclerosis (124), it still needs to be explored more in the context of T1D.

Recently, anti-CD40L antibody (frexalimab) has been under investigation in phase 2 clinical trials for preserving endogenous insulin production in newly onset T1D patients (60). Additionally, anti-CD40L has been explored for islet graft rejection mitigation. For instance, preclinical work by Anwar et al. demonstrated an engineered anti-CD40L antibody (tegoprubart) that reduces the risk of thromboembolic events for promoting islet survival in diabetic nonhuman primates (NHPs). Among two NHPs that received the islets and tegoprubart, one demonstrated long-term graft survival for 182 days and increased Treg frequency, while the other one rejected the graft after 2 weeks; however, it showed partial graft function after the second islet transplantation (118). All these promising studies with anti-CD40L lead to an ongoing clinical trial by Eledon in collaboration with the University of Chicago Transplant Institute for evaluating anti-CD40L to prevent rejection of transplanted islets (125).

Another commonly explored therapy for APC blockade is anti-CD20. CD20 is mainly expressed on the surface of B cells (**Figure 3F**) (126). Hence, anti-CD20 is called B-cell-depleting therapy (61). Previously, a phase 2 clinical trial with rituximab, an anti-CD20 monoclonal antibody, revealed that four infusions of rituximab over 1 month in recent-onset T1D patients delayed the reduction in C-peptide by 8.2 months (61). This encouraged another clinical trial in combination with autologous expanded Tregs to improve efficacy. However, although the combination with Tregs was better than the Treg alone group, the treatment did not significantly delay the T1D progression (62). Additionally, like in the previous study, there were significant adverse events, 93% in the first study (127) and 80% in the combination study (62), associated with the treatment.

#### 3.2.3 Cytokine blockade

Cytokines play a complex role in T1D pathogenesis, and inflammatory cytokines have been associated with the pathogenesis of T1D as they can exacerbate the condition (128, 129). Several cytokines, such as IFN-γ, IL-1β, tumor necrosis factor (TNF)-α (130–132), IL-6 (133), IL-8 (134), IL-12 (135), IL-21 (136), IL-23 (137) have been implicated in β-cell destruction. Several anti-cytokine therapies have been under investigation in clinical trials to address the negative effects of these pro-inflammatory cytokines (**Table 1**). One such therapy is anti-TNF-α, a monoclonal antibody (golimumab) that demonstrated a 43% lower reduction in C-peptide levels compared to control after 52 weeks of biweekly subcutaneous injections in phase 2 clinical trials in new-onset T1D youth (63). A 2-year follow-up study showed similar trends (64). However, 23% of the golimumab group experienced adverse events associated with the treatment (63). Another TNF-α-blocking therapy is etanercept, which binds to TNFα by acting like its receptor (**Figure 3G**) (138). The pilot trial, which used a combination of etanercept, GAD-alum, and vitamin D, showed that the treatment was well tolerated but failed to delay the progression of T1D in patients with recent onset (65). IL-6 receptor inhibitor (tocilizumab) is another recently investigated cytokine blockade therapy for new-onset T1D patients. A phase 2 trial with tocilizumab administered IV monthly for 7 months did not delay the loss of β-cells function or change frequencies of CD4 or Treg subsets (66). Another phase 2 clinical trial evaluating the IL-8 receptor inhibitor (ladarixin) administered twice daily in three cycles of two weeks on and two weeks off, in newly diagnosed T1D patients, failed to demonstrate efficacy in delaying disease progression (67). Both studies showed no adverse events associated with the treatment.

Another phase 2 trial evaluated the efficacy of the combination of anti-IL-21 antibody (**Figure 3I**) (NN-8828) and glucagon-like peptide-1 receptor agonist (GLP-1 RA), liraglutide, in adults with recent onset of T1D. Liraglutide was utilized as a GLP-1 RA, which has been shown to be effective in improving glycemic control in T1D patients (139). Anti-IL-21 was administered intravenously every six weeks, while liraglutide was given via subcutaneous injections once daily for 54 weeks. This combination resulted in 48% higher C-peptide levels and a 34% reduction in hypoglycemia in the treatment group after 54 weeks; however, these effects did not last after treatment was discontinued. Additionally, no changes in immune cells were observed (69). A liraglutide and *ex vivo* expanded Treg combination therapy is currently being assessed in a Phase 1/2 clinical trial. Ustekinumab, a monoclonal antibody targeting the p40 subunit shared by IL-12 and IL-23, was recently investigated in adolescents with recent-onset T1D (**Figure 3H**). Subcutaneous injections of ustekinumab at weeks 0, 4, and then every 8 weeks until week 44 resulted in 49% higher C-peptide levels than controls at 52 weeks and were well tolerated in patients. While these results are promising, the delayed efficacy of ustekinumab and anti-IL21 compared to less specific T-cell blockade therapies raises concerns about their ability to delay disease progression effectively before the complete loss of β-cell function (70).

Overall, immune blockade strategies have been shown to be efficacious in delaying diabetes onset in some patients with T1D. Furthermore, FDA approval of teplizumab furthered interest in targeting immune players involved in autoimmune attack against β-cells. Building on this momentum, other T cell and APC blockade monoclonal antibodies, initially developed for promoting graft tolerance, are now being repurposed to be investigated for T1D prevention. However, several limitations are associated with these approaches, such as systemic side effects, limited longevity of tolerance, and increased risk of immune-related complications. Although blockades of pro-inflammatory cytokines are currently being explored as an alternative, more targeted approach, the results from clinical trials show that inhibiting these proinflammatory signals may not be enough for robust and sustained control of autoimmunity.

Importantly, most clinical trials evaluate these therapeutics for patients at stage 3 of the disease, potentially missing the opportunity window for preserving more β-cell function. This highlights the need for more advanced and precise screening to identify at-risk individuals earlier and evaluate these interventions for earlier stages of disease. Looking ahead, utilizing regenerative approaches in combination with developing more targeted delivery methods may allow for safer, more durable, and more effective prevention of diabetes progression.

### 3.3 Cell-based therapies

Cell-based immune therapies have also emerged as a promising treatment for T1D. Therapies in this area seek to target cells involved in T1D pathogenesis and use them to induce tolerance in host immune systems. Tregs and tolerogenic dendritic cells (tDCs) have garnered particular attention in this area. Recent clinical studies have proven the safety of autologous Tregs in human patients (**Table 1**). However, a strong and desirable immune response was not generated through this method alone (71, 140). Combinatorial regimens and engineered Tregs are being tested in preclinical models to improve efficacy and tolerance. Similarly, tDCs exhibit significant immunoregulatory functions, promoting Treg induction and inhibiting proinflammatory responses. Clinical trials have proven the safety of autologous tDCs, with a current phase 1 clinical trial for T1D assessing a vaccine made of host dendritic cells and a proinsulin protein. Current efforts similarly focus on improving tDC efficacy through genetic engineering or combinatorial regimens in preclinical and clinical trials. Outside of tDCs and Tregs, other cell types such as platelets and MSCs have also been explored for localized immunomodulation. This section reviews recent efforts in cell-based therapies, including insights from clinical trials, engineering efforts, and combinatorial regimens, while addressing remaining challenges in translating these approaches to the clinic.

#### 3.3.1 Regulatory T cell therapy

T cells play a significant role in the inflammation and progression of T1D as cytotoxic T cells destroy pancreatic β-cells. An imbalance between effector T cells and regulatory T cells has also been observed in early T1D development before disease onset (141). Tregs can suppress autoreactive T cells, a critical T1D onset and progression component. For this reason, a depleted Treg population has been implicated in T1D pathogenesis (142, 143). Due to the anti-inflammatory nature of Tregs and their role in autoimmunity, they have been of interest for immunomodulatory therapies (144, 145). While Tregs are also explored for prolonging islet grafts, this section will mostly discuss their application in T1D prevention, where their therapeutic application has been more extensively pursued. Despite their therapeutic promise, Tregs can be hard to isolate from blood and other tissues, making it difficult for T-reg-based therapies to be viably used. Therefore, many therapies focus on engineering effector T cells into Tregs *ex vivo* to prevent the onset of T1D. Although Tregs are also explored for prolonging islet transplantation survival in clinical trials, this section will mostly discuss prevention effects as more efforts are done in this front.

Clinical trials have primarily been conducted with autologous polyclonal Tregs, which have been well tolerated in patients under a standard immunosuppressive regimen, supporting the safety of Treg therapies (140). Combining Treg therapies with low-dose IL-2 has also been tested in clinical trials. Low-dose IL-2 has been shown to induce an anti-inflammatory gene expression profile in T1D, selectively expand Tregs, and reduce the frequency of IL-21^+^ T cells (146). In a phase 1 trial by Bluestone et. al, T1D patients were treated with polyclonal Tregs and low-dose IL-2. The IL-2 increased endogenous Tregs within patients’ peripheral blood but also had an off-target effect and expanded cytotoxic cells. Although the Treg infusion was deemed safe, these authors stressed the importance of developing Treg therapies that are more specific to the patient Treg population and avoid off-target effects (71). This previous work can better inform future clinical trials of engineered Tregs and combinatorial regimens. In NOD mouse models, Tregs have also been used with other immunosuppressive agents such as cytokines, antibodies, and standard immunosuppressants to improve engraftment of donor Tregs (147, 148). Further, preclinical work in NOD mice has developed Treg engineering through chimeric antigen receptor technology and genetic engineering.

Chimeric antigen receptor (CAR) technology is a relatively new method to create Tregs for autoimmune diseases like T1D. Engineered Tregs using CARs allow for the redirection of Treg specificity towards specific targets, which is of special interest as antigen-specific Tregs have been more effective in preventing autoimmune disease onset than polyclonal Tregs (144, 149, 150). Using CAR Tregs to suppress the autoreactivity in T1D can help improve the efficacy of Treg-based therapies (151). Tenspolde et. al, for example, used CARs to change T cell specificity towards insulin and *FOXP3* transduction to redirect Teffs to Tregs. Although the generated Tregs did not prevent diabetes progression in NOD mice, they were still detectable after 4 months and mimicked the activity of natural Tregs. Their method was the first recorded instance in literature of using CAR technology to convert Teffs to Tregs for T1D (152). The MHC class II peptide complex has also been a target for CAR Treg technology. This is mainly due to previous work proving that Tregs can regulate immunosuppression by depleting MHC class II peptides (153, 154). Spanier et. al, for example, created the first T cell receptor-like CAR specific for an MHC class II peptide using a monoclonal antibody. In a NOD mouse model, T1D was suppressed entirely when treated with a 3:1 Treg/BDC2.5 T cell ratio (149). Zhang et al. showed that a single infusion of CAR T cells, MHC class II showed antigen-specific Tregs induced through this method, exert a greater protective effect than nonspecific polyclonal Tregs (155, 156). CAR T-regs have been used in clinical trials with some success, primarily as a therapy for solid tumors. While the targets for T1D CAR therapies are different and these previous trials utilize effector T cells rather than regulatory T cells, these examples show that CAR T cell therapy has been proven safe in humans and can likely be adapted for T1D treatment (157, 158).

Engineering gene expression is another method to engineer Treg specificity and T-reg mediated immunoregulation. In this area, many efforts focus on regulating Forkhead box protein 3 (*FOXP3*) expression, which is essential for Treg suppressive activity (159–161). Most recent studies in this area have used homology-directed repair (HDR) based gene editing to alter the expression of *FOXP3*. Gene editing through this method has been used to create stable and high expression of *FOXP3* in primary human CD4+ T cells, which displayed T-reg-like properties long-term and suppressed polyclonal islet-specific T cells from individuals with T1D and prevented diabetes onset in a T1D mouse model (162, 163). Uenishi et al. used similarly engineered Tregs enriched and expanded with rapamycin to prevent T1D in NOD mice, showing that engineered Tregs can be combined with standard immunosuppressants and enhance T1D outcomes (164). Outside of *FOXP3* expression regulation, Rafiqi et al. inhibited the eukaryotic initiation factor 5a (*eIF5a*), which is overexpressed in T1D patients and promotes a proinflammatory environment, as well as a key pathway in T cell maturation. A Treg phenotype was induced when this method was used on isolated human effector T cells. While these Tregs remain untested *in vivo*, the group has previously shown that this inhibition is safe in humanized mouse models and on islets *in vitro* (165, 166).

Clinical trials with Tregs have primarily served to verify the safety of autologous polyclonal Tregs in T1D patients. The focus of preclinical models has been engineering Tregs to enhance their specificity or manufacturing Tregs from Teffs cells. Current clinical trials have allowed for some testing of how autologous polyclonal Tregs are tolerated in patients, potential side effects, doses, and effective administration routes may vary for engineered Tregs. More broadly, concerns also include storage and complex manufacturing processes. In addition, Treg exhaustion can be a further point of concern that limits the efficacy of this therapy (167).

#### 3.3.2 Dendritic cell therapy

Dendritic cells (DCs) are another portion of the immune system being assessed to help form a method of regulating T1D. While there are DCs that tend to be more pro-inflammatory and have been implicated in the pathogenesis of T1D, there are other DCs more associated with immunosuppressive capacities, referred to as tolerogenic dendritic cells (tDCs) (168, 169). Tolerogenic dendritic cells are an immature to semi-mature form of dendritic cells and are functionally distinct from fully mature, pro-inflammatory DCs. Compared to proinflammatory DCs, they exhibit low expression of MHC class II and co-stimulatory molecules such as CD80, CD86, and CD40. Additionally, they are characterized by high expression of IL10 and TGF-β, as well as expression of PD-L1 and PD-L2 (170, 171).They can trigger immunosuppressive activity by influencing T cell populations, increasing Treg activity, and suppressing effector T cells (172–174). Further, tDCs are essential in regulating immune activity, and T1D onset has been associated with fewer tDCs in the pancreatic area (168, 175, 176). Therefore, therapies involving engineered tDCs present a promising avenue to T1D therapies.

Autologous DCs’ safety has previously been evaluated in humans, and current efforts in clinical trials focus on improving the therapy’s efficacy (177). The AVT001 autologous DC therapy targets the Q/E CD8+ Treg pathway, which is defective in many people with type 1 diabetes. This treatment was recently completed in a phase 1/2 clinical trial intended to evaluate the safety of the treatment in patients. Treatment was associated with less decline from normal C-peptide levels, but other indicators like HbA1c and insulin did not demonstrate significant change from baseline (75). However, these results indicate preservation of insulin secretion and show that DC therapies can be viable as T1D treatments. Another clinical trial, in phase 1, utilized vaccines with tDCs pulsed with a proinsulin peptide (73). After verifying this method’s safety, it could selectively stimulate Tregs and reduce effector T cell populations *in vivo*. This reduction was preserved even two years after treatment, and improved glycemic control was observed in eight of nine patients. These promising results suggest that this method can alter the immune system towards autoantigen-specific tolerance (74).

Preclinical work in NOD mice aims to further enhance the capabilities of tDCs to prevent the onset of T1D. For example, Passeri et al. created tDCs by genetically engineering monocytes with lentiviral vectors co-encoding for immunodominant antigen-derived peptides and IL-10. In two T1D mouse models, the engineered DCs completely prevented disease development in 38% and 45% of recipients by inducing Tregs and downregulating Teffs (178). Further, human dendritic cells engineered with this method were able to inhibit T effector cell responses and promote Treg populations in celiac disease patients *in vitro* (178). These results support the versatility of this method and its potential viability in clinical trials.

Current work in clinical trials has shown the safety and impact of autologous tDCs on the immune system of T1D patients. However, none of these trials exhibit high efficacy for T1D treatment. Current preclinical trials focus on engineering tDCs to improve effectiveness, with some promising results. However, barriers remain for adoption in clinical trials regarding doses, administration routes, and potential immunogenicity of engineered tDCs in humans.

#### 3.3.3 MSCs therapies

Other immune cells have also been explored to prevent the onset of T1D. MSCs, for example, have been a cell chassis of interest due to their immunomodulatory properties. MSCs have also been investigated in clinical trials as a treatment modality for T1D. Carlsson et. al’s 2014 study first verified the safety of MSCs in T1D patients, and more recent work has been focused on evaluating long-term safety and benefits of this method (179). In a phase 1/2 clinical trial by Izadi et. al, each patient received two doses of bone marrow-derived MSCs and was followed up with for one-year post-transplantation. MSC transplantation was safe long-term and shifted serum cytokine populations from proinflammatory to anti-inflammatory, emphasizing MSCs’ immunomodulatory effects (76). To provide more mechanistic analysis, Wu et al.’s preclinical study found that umbilical cord-derived MSCs helped improve C-peptide levels by inhibiting T cell proliferation (180). MSCs induce immune tolerance by suppressing autoimmune attack from effector T cells and shifting the immune environment to a more anti-inflammatory phenotype (e.g., through secretion of cytokines). Other studies have also noted MSCs’ ability to promote the generation and expansion of regulatory T cells and M2-like macrophages (181). MSCs have also been explored for islet transplantation. For instance, a study by Wang et al. demonstrated the use of engineered MSCs to express PD-L1 and CTLA4-Ig and co-transplanted them with islets in a T1D mouse model. Without systemic immunosuppression, the allograft survived for up to 100 days and had reduced infiltration by T-effector cells with a higher Treg presence (182). However, despite preliminary promising results, there is still a need for more effective and complete immunomodulation, which is the subject of exploration in many preclinical models. As with other therapies, patient-specific characteristics like lifestyle or autoantigens can further affect treatment efficacy, presenting an additional consideration when creating MSC-based therapies (183). Another consideration is poor MSC survival during transplantation due to host immune system attack, requiring long-term immunosuppression, and oxidative stress, among other factors (184).

#### 3.3.4 Platelet cell therapies

Another cell population of interest is platelets. Zhang et al. engineered platelets to express programmed death ligand 1 (PD-L1), a commonly explored immunomodulatory checkpoint ligand, achieving long-term reversal of new diabetes onset in 58% of 10-week-old NOD mice treated with ten IV injections of the modified platelets. Notably, these engineered platelets released platelet microparticles (PMPs), which exhibited enhanced infiltration into the pancreas due to their smaller size. Additionally, mice treated with the engineered platelets showed reduced pancreas-infiltrating CD8+ T cells and increased T regs within the pancreas (185). More recently, Zhang et al. bioengineered platelets to produce higher-than-normal amounts of immune checkpoint ligands, including PD-L1, PD-L2, B and T lymphocyte attenuator (BTLA), and Galectin-9 (Gal-9). With this technique, the amount and activity of pancreas-infiltrating T cells were reduced with an increase in Tregs and macrophage polarization towards the M2-like phenotype (186). Despite these results, the therapeutic potential of platelets is limited by their short lifespan, requiring frequent administration. Additionally, platelets may face targeting specificity and stability challenges, which could reduce their efficacy and increase off-target effects (187).

Many cells are involved in T1D and can be harnessed for cell-based therapies. We have discussed MSCs and platelets, including their work in clinical and preclinical models and their drawbacks. While MSCs have immunomodulatory capabilities, poor cell survival presents a drawback. For platelets, their short lifespan limits clinical applicability. Similar disadvantages must be considered with these and other cell types potentially employed in this space.

## 4 Cell replacement therapies

Despite significant advances in insulin therapy and glucose monitoring technologies, Type 1 diabetes (T1D) remains a lifelong disease characterized by progressive β-cell loss, glycemic instability, and a substantial risk of acute and chronic complications. Even with intensive insulin management, many patients experience hypoglycemia unawareness, glycemic variability, and reduced quality of life, highlighting the fundamental limitation of exogenous insulin in replicating the dynamic, glucose-responsive function of endogenous β-cells. As a result, restoring functional β-cell mass remains the only strategy with the potential to provide durable physiological glycemic control. However, current β-cell replacement approaches are restricted to a small subset of patients with severe disease, require lifelong immunosuppression, and face significant barriers related to graft survival, immune rejection, donor availability, and long-term durability. These limitations underscore the urgent need to improve β-cell replacement therapies beyond existing clinical protocols (188–190).

Allogeneic islet transplantation replenishes the lack of functional β-cells in patients with T1D. The Edmonton protocol, which is the main procedure for transplanting donor human islets by infusion into the hepatic portal vein of the recipient, played a revolutionary role in the islet transplantation field since the early 2000s. Long-term follow-ups in patients receiving human islets have shown promising results in terms of better insulin independence for up to 20 years after transplantation (191–194). As a result, the U.S. Food and Drug Administration approved Lantidra by CellTrans, the first allogeneic (donor) pancreatic islet cellular therapy made from deceased donor pancreatic cells for treating type 1 diabetes. Lantidra is approved for the treatment of adults with type 1 diabetes who are suffering from severe hypoglycemia. The procedure involves administering 5000 islet equivalents/kg body weight via transhepatic access into the hepatic portal vein. 63% of the patients met the efficacy endpoint determined by HbA1c levels ≤6.5% and absence of severe hypoglycemic events (SHEs) through 1 year after last transplant (195). However, some critical challenges still need to be solved, such as a regulatory framework, like manufacturing consistency, or long-term efficacy. Additionally, transplanted islets are susceptible to instances of blood-mediated inflammatory reactions and rejection, which could lead to their early destruction. Graft rejection can occur through direct recognition, where host T cells identify donor antigens, or indirect recognition, where host antigen-presenting cells (APCs) present donor islet antigens to host T cells alongside costimulatory signals. Alloantigen-specific T cells are activated in both scenarios, destroying donor islets by CD8+ T cells (196–198).

Another point of consideration is that selecting an appropriate transplantation site for donor islets can significantly affect the efficacy of the transplanted cells. The most common transplantation site is the intrahepatic vein due to its accessibility and favorable blood supply. It allows fast diffusion of oxygen and nutrients to the cells and insulin into the blood system. However, this site also poses several challenges, such as instant blood-mediated inflammatory reaction (IBMIR), which leads to immediate loss of a significant portion of transplanted islets, reducing the overall efficacy of the procedure (199). IBMIR is caused by direct exposure of islets to the bloodstream, triggering pro-inflammatory cytokines to be released, which is followed by the activation and recruitment of innate immune cells, enhancing the inflammation and destruction of islets (200). Additionally, transplantation into the intrahepatic vein requires immunosuppressants as the liver’s immune environment can contribute to alloimmune and autoimmune responses against the transplanted islets. Another implantation site is the subcutaneous space, which is also easily accessible but has a relatively poor blood supply, which can impair islet survival and function (201). Finally, transplanting islets into the mesenteric fat offers a better vascularized environment than subcutaneous tissue. Nonetheless, this approach is still under investigation, and long-term outcomes remain uncertain (201).

In contrast, transplantation under the kidney capsule is widely used as a preclinical benchmark site, as it provides a confined and well-vascularized environment that supports robust islet engraftment and facilitates mechanistic studies of revascularization and immune responses. Nevertheless, the kidney subcapsular space has important limitations for Type 1 diabetes therapy, including poor clinical translatability, limited volume, a lack of physiological insulin drainage into the portal circulation, and continued susceptibility to autoimmune and alloimmune attacks in the absence of immunomodulation (202).

Cell therapies have been developed to overcome some of the limitations of using donor islets. One such example is Vertex’s ongoing phase 3 clinical trial using stem cell-derived islets to achieve glycemic correction in patients with T1D (**Table 2**). This trial investigates zimislecel (formerly known as VX-880), which involves the delivery of the insulin-producing cells via infusion into the hepatic (liver) portal vein. This approach demonstrated insulin independence in 10 out of 12 participants for 1 year (210). However, this platform still requires ongoing immunosuppression, using tacrolimus and mycophenolate mofetil (MMF) to suppress T-cell activation and inhibit lymphocyte proliferation, respectively, and therefore to ensure the immune cells do not attack transplanted cells. The need for immunosuppression drugs for life comes with several risks, such as developing kidney diseases (218, 219), infections (220), cancer (221), or β-cells toxicity-related issues (19). These issues highlight the need to develop novel approaches that can improve islet transplantation.

**Table 2:** Strategies for islet replacement.

Therapy type	Target/ additional information	Treatment name(s)	Progress	Limitations	Current status	Reference, clinicaltrials.gov identifier
Encapsulation	Engrafted pancreatic endoderm cells become islets	VC-02 (stem cell-derived pancreatic endoderm cells)	Safe, positive C-peptide levels in some patients	Only 35% of initial islet mass survived	Phase 1/2 completed 2023	(203), NCT03163511
Encapsulation	Subcutaneous implant, without immunosuppression and with oxygen supply	Human islets in Beta Air device	Results not posted	Results not posted	Phase 1/2 in progress	(204), NCT02064309
Implantation Device	Abdominal wall pre-vascularized device for 3 weeks with immunosuppression regimen	Sernova Cell Pouch	Stimulated C-peptide levels for 9 months; persistent graft function; reduced HbA1C and insulin dosage	Currently only 7 patients tested; preliminary results; invasive surgery	Phase 1/2 recruiting	(205), NCT03513939
Implantation Device	Subcutaneous abdominal implantation of pre-vascularized device for 22–130 days before islet transplantation	Sernova Cell Pouch	Results not posted	Results not posted	Phase 1/2 terminated	(206), NCT01652911
Implantation Device	Perforated device to deliver cells	VCTX210A, VCTX211 (devices to deliver genetically engineered allogeneic pancreatic endoderm cells)	Results not posted	Results not posted	Phase 1 completed (VCTX210A); Phase 1/2 in progress (VCTX211)	(207, 208), NCT05210530, NCT05565248
Implantation Device	Immunoprotective device that does not require additional immunosuppression	VX-264 (encapsulated stem cell derived islet therapy)	Trial stopped early due to lack of efficacy	Lack of efficacy led to termination	Phase 1/2 terminated (update posted 2025)	(209), NCT05791201
Stem Cell Derived Therapy	Hepatic Portal Vein infusion	Zimislecel (VX-880)	12 participants treated; restored endogenous insulin secretion; 92% insulin reduction; 10/12 insulin-independent at 1 year; HbA1c <7% and >70% time in range	Requires immunosuppression; durability beyond 1 year still uncertain	Phase 3 recruiting following successful Phase 1/2	(210, 211), NCT04786262
Stem Cell Derived Therapy	Intramuscular transplantation	Hypoimmune islets	First-in-human treated July 2025; C-peptide detected; insulin production confirmed; HbA1c improved; effect ongoing at 6 months	N=1; short follow-up; donor islet supply constraints; long-term safety unknown	Phase 1 recruiting	(194, 212) NCT06239636
Monoclonal antibody	CD40 on antigen presenting cells	Tegoprubart (anti-CD40L)	Promising results in preclinical model	Results not posted	Phase 1/2 trial recruiting	(125), NCT06305286
MSC + Islets	Transplantation of islets and MSCs in T1D patients	Allograft islets and autograft MSCs	Results not posted	Results not posted	Current status unknown	(213), NCT00646724
Polyclonal Tregs	CD4+ T cells	Ex vivo-selected, and ex vivo-expanded autologous polyTregs combined with islet transplantation	Results not posted	Results not posted	Phase 1 in progress	(214), NCT03444064
Islet + Treg cells	Tregs from either donor or recipient administered with islets	Clinical Islet Transplantation with Apheresis, Isolation and Reintroduction of Recipient Regulatory T Cells	Results not posted	Results not posted	Phase 1 in progress	(215), NCT05973734
Polyclonal Tregs	CD4+ T cells	cePolyTregs (cryopreserved PolyTregs)	Results not posted	Results not posted	Phase 1 in progress	(216), NCT05349591
Encapsulation/Xenotransplantation	Encapsulated porcine islets + device for patients with established T1D and severe hypoglycemia	OPF-310	Phase I/II trial began; testing safety & dose (6000 or 12,000 IEQ/kg)	Early stage; unknown long-term safety, immune reaction, encapsulation device durability; supply/scaling of xenogeneic islets	Phase I/II recruiting/ongoing	NCT06575426 (217)

### 4.1 Islet engineering for enhanced immune evasion

In addition, to address some of the previously mentioned issues, several groups are trying to engineer islets to enhance immune evasion while keeping the functionality of donor islets. One of these strategies is the use of hypoimmune islets, which are genetically engineered pancreatic islets designed to evade immune system detection and rejection. The use of hypoimmune islets allows for the elimination of lifelong immunosuppression while achieving glycemic correction. Hu et al. developed human allogeneic gene-engineered hypoimmune islets, which showed glycemic correction in pre-clinical trials. The authors achieved hypoimmunity by eliminating MHC class I and II expression by knocking out the genes β2-microglobulin (*B2M*) and class II major histocompatibility complex transactivator (*CIITA*), respectively, using CRISPR-Cas9 gene editing techniques. In addition, they overexpressed CD47 to prevent phagocytosis by macrophages and natural killer (NK) cell-mediated destruction. The findings demonstrate that the hypoimmune (HIP) islets evaded rejection and restored normal blood glucose in allogeneic humanized mice, autoimmune NOD mice, and STZ-induced diabetic mice for at least 30 days (222). In addition, this same group applied their HIP modifications to induced pluripotent stem cells (iPSCs) and tested their survival in a fully immunocompetent, allogeneic rhesus macaque model without immunosuppression. The key findings showed that HIP-iPSCs survived for over 16 weeks after transplantation, significantly longer than unmodified iPSCs, which were rapidly rejected. These HIP cells successfully differentiated into multiple cell types without triggering immune activation, confirming their ability to evade both innate and adaptive immune responses (223). Additionally, the same team also similarly engineered primary pancreatic islets derived from rhesus macaques to create “hypoimmune pseudo-islets”. The authors implanted the engineered cells into a fully immunocompetent diabetic cynomolgus monkey without administering immunosuppressive drugs and showed significant improvements in glucose regulation by observing an initial increase and later stabilization in serum C-peptide levels correlated with controlled blood glucose levels (224). The technology derived from these findings is currently undergoing phase 1 clinical trials as the first in-human safety study of hypoimmune pancreatic islet transplantation in adult subjects with type 1 diabetes (**Table 2****) (**225).

On the other hand, Parent et al. followed a different approach by modifying human pluripotent stem cells (hPSCs) into insulin-producing cells to minimize the immune system’s recognition and rejection of these cells, by deleting specific HLA genes responsible for presenting antigens to immune cells. The authors removed the highly polymorphic *HLA-A*, *HLA-B*, and *HLA-C* genes, except for retaining the common *HLA-A2* allele and the less variable *HLA-E*, *HLA-F*, and *HLA-G* genes. By using humanized mouse models, the researchers demonstrated that these genetically altered islet cells experienced significantly reduced NK cell activity and diminished T-cell-mediated alloimmune responses while not impairing the cells’ ability to function as insulin-producing β-cells (226). Similarly, Gerace et al. genetically engineered human stem cell-derived islets (SC-islets) to express PD-L1 and HLA-E, two immune-regulatory molecules that inhibit T cell and NK cell responses. Using CRISPR-Cas9, the authors knocked out HLA class I molecules to reduce allogeneic recognition while overexpressing PD-L1 to engage inhibitory PD-1 receptors on T cells. The engineered islets were encapsulated in a retrievable device and implanted into immunocompetent mice. These modifications allowed the islets to evade immune rejection and maintain function without systemic immunosuppression.

### 4.2 Islet encapsulation in micro or macro devices to evade immune clearance

Encapsulation of islets in a biomaterial has previously been shown to be promising in preclinical studies for the long-term survival of transplanted islets by providing an immune-isolative layer to avoid direct graft recognition without immunosuppression (203, 227–231) (203, 228–232) (203, 227–230, 233). Additionally, microencapsulated stem-cell-derived β-cells have been evaluated in clinical trials. Shapiro et al. initially developed a macro-device made of a multi-layer platform made of expanded polytetrafluoroethylene with engineered portals and an external polyester mesh that provides stability. Pancreatic endoderm cells (PEC) were then housed within the macro-encapsulation device, which provided immune protection from the host (VC-02) (**Table 2**) (203). This platform was tested in a phase 1/2 clinical trial for T1D and demonstrated positive C-peptide levels in 35,3% of patients as early as 6 months post-implant. The device was implanted subcutaneously under the immunosuppression regimen initiated with ATG and maintained with, mycophenolate mofetil and tacrolimus, common immunosuppression drugs to avoid transplant rejection. This clinical trial showed the potential of microencapsulation devices to house β-cells for immune protection and correction of blood glucose levels in type 1 diabetes patients. Despite promising outcomes, analysis of retrieved devices revealed that only 35% of the initial islet mass survived, a significantly lower percentage compared to preclinical studies. This discrepancy may be attributed to a more robust foreign body response in humans, among other contributing factors (231). These findings suggest that current immune isolation strategies, such as microencapsulation and standard immunosuppression regimens, are insufficient to fully mitigate the foreign body response, thereby limiting the long-term survival of transplanted islets. The implantation of foreign materials triggers a cascade of responses that leads to inflammation and recruitment of the immune cells at the site of the implant. This process results in a dense fibrous capsule formation around the device. This fibrotic barrier hinders the exchange of essential nutrients and oxygen, as well as the diffusion of insulin, ultimately compromising the viability of implanted islets and limiting the functionality and longevity of the implant (232, 234, 235).

In parallel, Sernova Biotherapeutics has developed a promising encapsulation platform known as the Cell Pouch™ System, which is an implantable, vascularizing device engineered to house and support insulin-producing cells. So far, for the ongoing phase 1/2 clinical trial at the University of Chicago, the Cell Pouch™ has shown encouraging results in patients with type 1 diabetes who suffer from hypoglycemia unawareness (**Table 2**). In Cohort A, which involved the implantation of the 8-channel Cell Pouch followed by donor islet transplantation, all six patients achieved sustained insulin independence, with durations ranging from 5.5 months to over four years. The first patient maintained insulin independence for more than 50 months, with HbA1c levels consistently within the non-diabetic range (≤6.5%). Histological analyses confirmed the presence of well-vascularized and functioning islets, producing insulin, glucagon, and somatostatin, even five years post-transplant. In the newer Cohort B, featuring an upgraded 10-channel Cell Pouch with 50% more islet capacity, preliminary data from seven patients show continued production of C-peptide and improved HbA1c levels, suggesting effective and durable islet function. The device has been well-tolerated across all participants, with no significant safety concerns reported, further supporting the potential of the Cell Pouch as a long-term, scalable therapy for restoring natural insulin production in T1D patients (236).

On the other hand, to further enhance the clinical translatability of encapsulation platforms, another approach utilizing oxygen-generating or supplementing technologies is explored. This strategy allows for the survival of high-density encapsulated islets, which can be beneficial for reducing implant size. For instance, Evron et al. demonstrated a device housing encapsulated islets within an alginate protected by an outer polytetrafluoroethylene (PTFE) membrane, thereby shielding them from host immune responses. These encapsulated islets were supplied with oxygen through the adjacent chamber, which was replenished daily through an external port. This device allowed for the survival of encapsulated allogeneic islets at a surface density of 4,800 islet equivalents/cm^3^ for more than 7 months in diabetic rats (237). This device is currently investigated in clinical trials (238). Another study by Wang et al. utilized an oxygen-generating device that recycles carbon dioxide generated by islets and converts it into oxygen via lithium peroxide particulates. Here, islets were encapsulated in alginate hydrogel and connected via a hollow silicon tube to the gas tank containing the PFC-immersed lithium oxide formulation. This approach allowed for the survival of rat islets in the diabetic mouse model for over three months (239). Krishnan et al. developed a different oxygen-generating method using electrochemical water splitting by proton-exchange membrane electrolysis to achieve functional survival of encapsulated rat islets at ~1,000 islets/cm^2^, when implanted in the subcutaneous space of diabetic mice for over 1 month (240). A different approach by Pham et al. utilized body moisture via electrolysis to support a high density of 60,000 IEQ/mL islets. This device allowed for survival of allogeneic islets for up to 3 months in a diabetic rat model (241).

### 4.3 Immunomodulatory biomaterials

Building on the promise of encapsulation devices, immunomodulatory biomaterials are being developed to enhance graft survival, minimize immune rejection, and avoid systemic toxicity associated with traditional immunosuppression. These materials can create a localized immune-privileged site through foreign body evading-chemical modifications, sustained release of cytokines or chemokines, presentation of checkpoint ligands, or localized delivery of immunosuppressive drugs. This section outlines the diverse strategies used to modulate the local immune environment, their application in preclinical studies, and challenges that remain for durable and scalable translation for curing T1D.

#### 4.3.1 Chemically modified biomaterial for evading foreign body response

**Figure 4:**
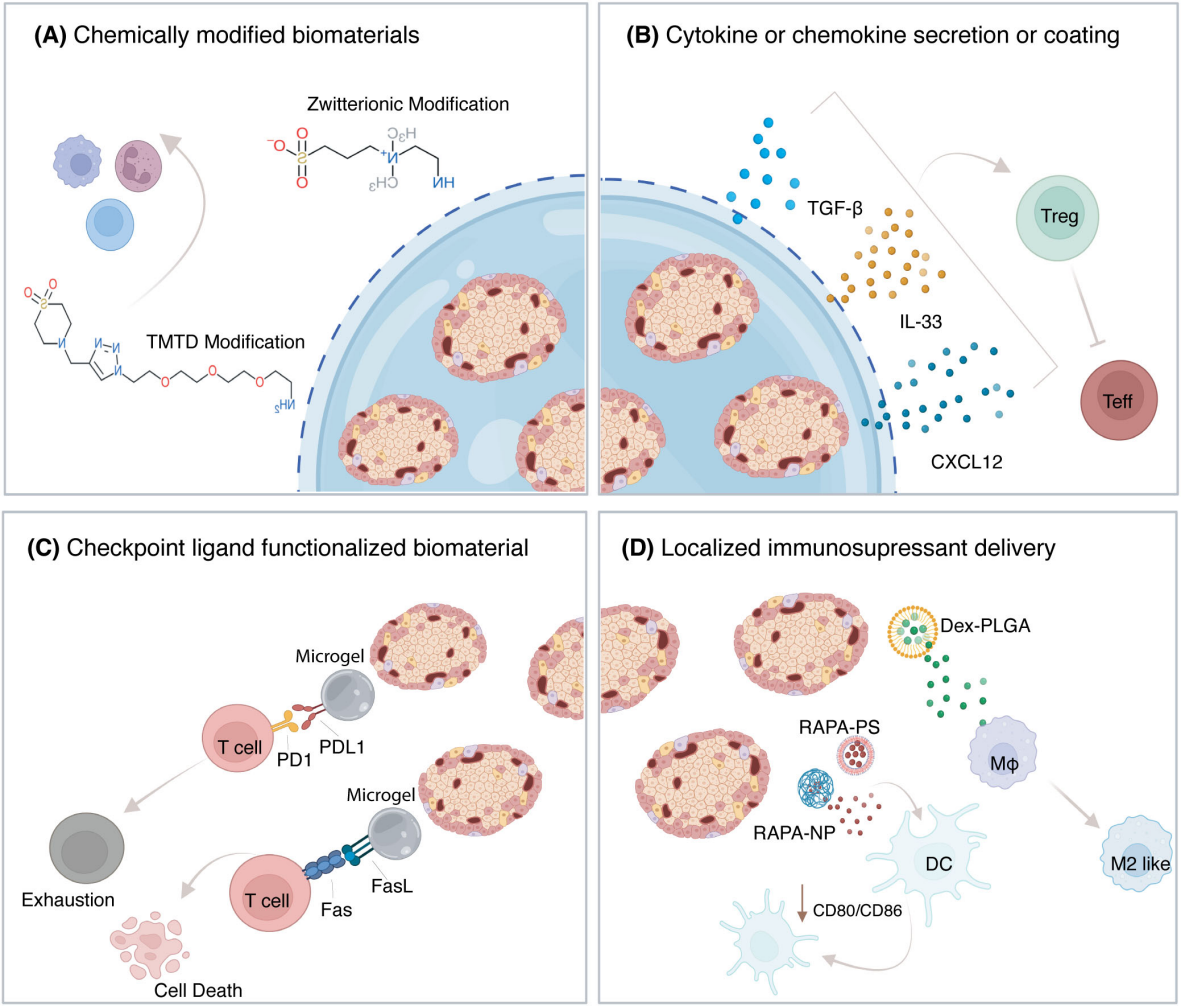
Immunomodulatory biomaterial strategies to prolong islet graft survival. **(A)** Chemical modification of the biomaterials can allow for prolonged evasion of immune recognition and subsequent fibrosis of the implant. **(B-C)** Biologics can be conjugated to a biomaterial or encapsulated within the material for direct presentation or localized release to immune cells in the vicinity. **(B)** Incorporation of anti-inflammatory cytokines such as IL-33, TGF-β, and CXCL12 within the biomaterial device can allow for a localized shift of T cell phenotype to Treg phenotype to suppress effector T cells. **(C)** While checkpoint ligands presented on the biomaterial can allow for inhibition of T cell activity by leading to their anergy or Fas-mediated death. **(D)** Additionally, polymer micro/nanoparticles can be co-delivered with islets for the release of immunosuppressants locally to the islet engraftment site. Mechanistically, these strategies primarily aim to limit cell adherence and inflammatory cell proliferation and coax cells in the surrounding area towards an anti-inflammatory lineage. For instance, delivery of Dex-PLGA microparticles leads to an M2-like shift in macrophages while nanoparticle delivery downregulates CD80/86 in dendritic cells, which can lead to suppression of immune responses. Treg, regulatory T cell; RAPA-PS: rapamycin polymersome, RAPA-NP: rapamycin eluting nanoparticle, Dex-PLGA: dexamethasone eluting PLGA micelle.

To avoid the use of immunosuppressants and allow for low immune recognition during islet transplantation, research has focused on chemical modifications to materials that reduce immune recognition (**Figure 4A**). For example, Vegas et al. encapsulated human embryonic-derived β-cells with triazole-thiomorpholine dioxide (TMTD) modified alginate and implanted them in the intraperitoneal space of C57BL/6J mice, which allowed for 174 days of normoglycemia (227). The same approach has been demonstrated in a non-human primate by implanting encapsulated allogeneic islets in the omental bursa of macaques, where islets remained viable for up to 4 months (229). Zwitterions are another class of molecules that have been extensively studied for their antifouling properties (242–244) and, more recently, for their use as a biomaterial chemical modification for preventing the foreign body response. Zwitterions prevent fibrosis by forming a strongly hydrated, charge-balanced surface that resists protein adsorption and immune cell adhesion. This blocks the activation of macrophages and fibroblasts, interrupting the fibrotic cascade at its source. Complementing these passive surface modification strategies, next-generation drug-releasing systems have been developed to actively achieve localized immunomodulation. For instance, co-encapsulation strategies utilizing crystallization-analyzed drug-eluting materials can provide sustained, low-dose release of anti-inflammatory agents for months to years. This approach has demonstrated prolonged islet graft survival while maintaining systemic drug levels below toxicity thresholds, effectively mitigating the risks of off-target effects (245). In addition, Liu et al. achieved long-term glycemic control for 200 days in STZ-treated diabetic mice using zwitterionic sulfobetaine-modified alginate and xenogeneic islets (246). Building on their success with fibrosis-evading zwitterions, they further enhanced their device by incorporating polyurethane for improved mechanical strength (247). Rat islets suspended in alginate and encapsulated within a polyurethane-zwitterion polymer and implanted in the IP space of C57BL/6J mice for six months demonstrated a reduction in (17.3% & 21.1%) insulin and fibrinogen adherence, compared to a non-modified control (113). TMTD modification was directly compared with zwitterionic material modification by coating a xenogeneic cell encapsulation device with zwitterionic coatings, poly(phosphorylcholine), poly(carbxybetaine), poly(sulfobetaine), or TMTD coating tetrahydropyran phenyl triazole and implanting in STZ-C57BL/6 mice (248). TMTD devices allowed for longer normoglycemia (60 days vs 4 weeks) and less surface collagen content at 4 weeks compared to zwitterionic-modified devices.

#### 4.3.2 Localized delivery or coating with anti-inflammatory cytokines and chemokines

Another strategy to reduce immune response for islet transplantation is the use of immunomodulatory molecules like cytokines and chemokines, which can be incorporated into microdevices to create a localized tolerogenic environment that supports encapsulated islets (**Figure 4B**). By selecting biomaterials with varying diffusion rates, the release of these molecules can be controlled and sustained over time, ensuring prolonged therapeutic effects through material biodegradation (249). For example, Chen et al. incorporated the chemokine stromal cell-derived factor-1α (CXCL12), which can repel effector T cells while recruiting Tregs into alginate microcapsules to support long-term allo- and xenoislet transplantation without systemic immune suppression. The authors investigated two distinct incorporation methods (1): direct surface coating (adsorption) of CXCL12 onto the islet surface, and (2) matrix co-encapsulation, where soluble CXCL12 was homogenously mixed into the alginate hydrogel precursor prior to crosslinking abreat concentrations ranging from 100 ng/ml to 1 µg/ml. The co-encapsulation of CXCL12 and mouse islets into microcapsules led to delayed rejection of both allogeneic and xenogeneic islets transplanted into the peritoneal cavity of diabetic C57/BL6 mice, enabling a linear release of CXCL12 over 25 days (250). Similarly, Alagpulinsa et al. encapsulated stem-cell derived β-cells with CXCL12 and showed that a concentration of 2.0 µg/ml CXCL12 can enhance long-term glucose responsiveness and glycemic correction in STZ-induced diabetic mice for 150 days. Correction of blood glucose levels was facilitated by preventing foreign body response and prolonging cell viability when encapsulating high concentrations of CXCL12 (251). CXCL12 has also been used in NHP models. Sremac et al. incorporated this chemokine into alginate microbeads to evaluate the post-implantation immunological and histopathological changes over 6 months. Implantation of 400,000 alginate beads with CXCL12 showed a lower basal activation state for cytokine levels than the NHP without CXCL12. Additionally, granulocyte-macrophage colony-stimulating factor (GM-CSF), IL-18, IL-5, and IL-6 showed significant variation in concentration between different time points for the CXCL12 and control groups. Overall, these results provided support for the utility of CXCL12 in immunomodulation for these types of alginate microbeads (252). In addition to CXCL12, other immunomodulatory factors have also been combined with biomaterials to extend their release over time.

Transforming growth factor-β1 (TGF-β1) has previously been co-delivered with islets within microporous poly(lactide-co-glycolide) (PLG) scaffolds, showing controlled release of the protein over 30 days. The addition of TGF-β1 showed delayed graft failure when transplanting allogeneic islets from Balb/c mice into diabetic C57BL/6 mice (253). Another study by Neshat et al. demonstrated artificial APCs that are made of microparticles (700 +/- 200nm) of poly(lactic-co-glycolic acid/poly(beta-amino ester)) (PLGA/PBAE) with anti-CD3 and anti-CD28 antibodies conjugated and TGF-β loaded within for co-transplantation with islets to activate T\reg cells local to the transplant site. Subcutaneous injection of allogeneic islets and artificial APCs in STZ-induced C57BL/6 mice as a proof of concept revealed increased FOXP3 (Treg) expression 3 days after implantation and improved glucose correction compared to controls within 20 days (254). Though long-term glucose correction was not demonstrated, the artificial APCs can be optimized and delivered in combination with other strategies for improved graft survival. Another study demonstrated that the use of IL-33, an M2-polarizing cytokine, was added to PLG scaffolds, which allowed a delay in early engraftment of allogeneic islets and diabetes reversal compared to control scaffolds. Further research showed that IL-33 presence in the allogeneic model enriched counts of beneficial Treg cells, shifting their percentage in the population of total CD4^+^ cells from 40% to 75%, while also decreasing graft-rejecting CD8+ cells (255).

#### 4.3.3 Localized checkpoint ligand presentation or release

Another widely explored strategy for creating a localized tolerogenic environment using biomaterials involves the presentation or controlled release of checkpoint ligands from micro- or macro-devices, which engage inhibitory receptors (such as PD-1 or CTLA-4) on T cells to suppress their activation, promote regulatory T cell induction, and ultimately establish immune tolerance at the implantation site (**Figure 4C**). One notable example is the NICHE (Neovascularized Implantable Cell Homing and Encapsulation) device developed by Paez-Mayorga et al. This macro-device features a dual reservoir system housing vascularizing mesenchymal stem cells (MSCs) and rat islets, while the outer reservoir contains CTLA-Ig and anti-lymphocyte serum (analog of rat ATG) for local delivery was implanted in the subcutaneous space of STZ diabetic eight-week-old male Lewis rats. In combination with rat islets, the NICHE device maintained normoglycemia for over 175 days, requiring periodic refilling of the drug reservoir on days 0, 28, and 56 (230). These results are obtained due to the favorable immune profile displayed by the treatment, exhibiting a significantly reduced number of cytotoxic T cells and macrophages, as well as pro-inflammatory cell types. Moreover, in NHP biocompatibility studies, the authors showed a 4-fold decrease in infiltrating leukocytes when the NICHE device was present. In summary, this macro-device platform shows promising results in modulating the immune system for islet transplantation; however, re-administration of both islets and local immunosuppressants was still needed to achieve the exhibited results (230). This technology has been licensed by NanoGland LLC and is in the initial stage of the FDA regulatory pipeline (256).

CTLA4-Ig has also been incorporated into other biomaterial platforms for localized delivery. For instance, Kuppan et al. developed CTLA4-Ig-loaded microparticles to induce long-term survival and tolerance of murine islet allografts by co-transplanting them with PLGA microparticles loaded with cyclosporine A (CsA) and a short course of CTLA4-Ig. CsA is a potent immunosuppressive drug used to prevent allograft rejection in solid organ transplantation and to treat autoimmune diseases. Co-delivery of BALB/c mice islets with CsA PLGA microparticles and CTL4-Ig into C57BL/6 mice led to about 55% of the recipients maintaining allograft function for 214 days post-transplant. Further study of proinflammatory cytokine profiles at 7 days posttransplant showed IL-1β, IL-6, INF-γ, and TNF-α expression reduced in both recipients treated by CsA microparticles and those treated by CsA microparticles and CTLA4-Ig compared to islet-alone recipients (257). In contrast, Barra et al. conducted a study showcasing the efficacy of non-toxic poly(N-vinylpyrrolidone) (PVPON) coated with antioxidant tannic acid (TA) and CTLA4-Ig, used to encapsulate NOD.Rag islets and implanted into the kidney capsule of C57BL/6 mice. The PVPON/TA/CTLA4-Ig encapsulated islets demonstrated remarkable graft survival exceeding 125 days. Furthermore, this approach effectively reduced proinflammatory macrophage signaling via diminished STAT1 activity, suppressed effector T cell populations, and promoted an increase in Tregs and anergic CD4+ cells, highlighting its potential to modulate immune responses favorably (258).

Programmed death ligand 1 (PD-L1) is another checkpoint ligand that is commonly incorporated within biomaterials for localized tolerance. PD-L1 binds to the inhibitory receptor PD-1 expressed at the surface of activated T cells. This interaction suppresses T effector function, playing a crucial role in maintaining immune homeostasis and promoting tolerance (259, 260). Several studies on T1D patients reveal abnormalities in the PD-1/PD-L1 pathway, suggesting it is essential for maintaining tolerance within the pancreas (259). Coronel et al. employed microgels made of four-armed poly (ethylene glycol) PEG macromers conjugated to streptavidin-PD-L1 (SA-PD-L1), which were co-transplanted with BALB/c mouse islets from in the epididymal fat pads of C57BL/6 STZ-induced mice. A low-dose 15-day course of rapamycin, which is an immunosuppressive drug that inhibits the mTOR pathway to directly inhibit T cell proliferation (261), was given to some groups of mice to mitigate an immune attack on the graft during the early post-transplantation period. The group of mice that received SA-PD-L1 microgels only demonstrated that 22% of grafts survived, with mice staying normoglycemic for 50 days. While, the mice that received a course of rapamycin, in addition, showed that around 60% of grafts survived for over 100 days (262). Gene expression analysis revealed an increase in regulatory factors *FOXP3*, *Jun*, *Egr3*, *Ccr4*, and *PD-1*, and flow cytometry showed an increase in Treg cells in response to SA-PD-L1 microgels. Further, PD-L1 microgels were shown to affect innate immunity by increasing macrophages at the transplant site. In this study, epididymal fat pads were selected as the implantation site due to their similarity to the murine equivalent of the omentum (263). The omentum has been demonstrated to be a superior site for islet transplantation, as it supports significantly better revascularization compared to the kidney capsule (264). In addition, Colonel et al. evaluated the combination of PD-L1 presenting microgels with a short course of the immunosuppressant rapamycin for the prevention of islet rejection in autoimmune NOD mice. Results showed significant extended islet graft survival due to microgels, with approximately 60% of recipients maintaining normoglycemia for over 100 days. Furthermore, the therapy promoted a tolerogenic immune environment at the graft site. There was an increase in regulatory T cells (CD4^+^CD25^+^FoxP3^+^) and a rise in exhausted cytotoxic T cell populations, which are associated with reduced immune-mediated graft destruction (265).

Fas ligand (FasL) is another commonly studied checkpoint ligand in this context. FasL binds to Fas on activated T cells, inducing apoptosis (266), and is essential for eliminating autoreactive lymphocytes (267). Previously, the same team that developed SA-PD-L1 microgels demonstrated using the same platform with FasL for the long-term survival of allogeneic islets with a short course of rapamycin in the kidney capsule of STZ-induced C57BL/6 mice for more than 200 days (266). This success inspired further studies in diabetic NHPs, where SA-FasL microgels were co-transplanted with allogeneic islets into the omentum under transient rapamycin treatment. These NHPs maintained normoglycemia for over six months. The omentum was selected as the implantation site to avoid thrombosis risks associated with portal vein delivery of microgels and islets, further demonstrating its translational potential (268). Li et al. conducted another study utilizing SA-FasL, but for modifying PLG scaffolds encapsulating β-cells derived from human pluripotent stem cells. These scaffolds were implanted into the epididymal fat pad of NSG mice and retrieved for analysis on days 4, 7, and 14 post-transplantations. The results showed significantly reduced infiltration of neutrophils and macrophages in comparison to the control group, highlighting the immune-modulatory potential of this approach (269).

#### 4.3.4 Localized immunomodulatory agent delivery

Another approach to prevent the rejection of transplanted islets involves the use of immunomodulatory agents, which help regulate the immune system’s response to donor islets. To minimize the side effects associated with systemic immunosuppression, these agents have been incorporated into biomaterial delivery platforms, allowing for localized administration (**Figure 4D**). Kuppan et al. were able to fabricate dexamethasone (Dex) -eluting PLGA micelles to increase immune protection in a murine islet allograft model. Dex is a corticosteroid that has the ability to promote the conversion of macrophages into their more favorable anti-inflammatory M2-like phenotype (270). Dex PLGA micelles were characterized to have the cumulative release of dexamethasone at different concentrations for over 30 days. When combined with CTLA4-Ig and transplanted islets into a diabetic mouse model, 80% (8 of 10) of the recipients maintained allograft function for 60 days posttransplant, doubling the long-term normoglycemia prevalence compared to CTLA4-Ig monotherapy recipients (271).

Moreover, biomaterials can be integrated with immunomodulatory agents to reduce the immune response when transplanting β-cells for T1D. Burke et al. loaded rapamycin into poly(ethylene glycol)-b-poly(propylene sulfide)) (PEG-b-PPS) polymersomes (rPSs). Authors were able to achieve encapsulation efficiency over 55%, which, when implanted in the subcutaneous (SC) space of C57BL/6 mice it led to a potent immunomodulation response compared to unloaded polymersomes. Further studies analyzed mouse organs (blood, liver, spleen and lymph nodes) after implantation of rapamycin-loaded rPSs at different time points, showing downregulation of CD80 and CD86 coreceptors in DCs in addition to a significant decrease in CD4 expression. These immunosuppressive abilities enabled a reduction in the effective drug dosage needed to achieve normoglycemia in STZ diabetic C57BL6 mice for 100 days by receiving 200 IEQs from BALB/c mice into the liver via the portal vein (261). Similarly, Nguyen et al. combined MSCs with rapamycin-releasing PLGA microparticles (RAP-MPs) to prevent immune rejection of islet xenografts in diabetic C57BL/6 mice. When transplanting 400 rat IEQs with and without the RAP-MPs spheroids into the liver capsules of STZ-induced C57BL/6 mice, recipients administered with 100 ng of RAP (HS100) within the spheroids led to normoglycemia for up to 60 days. Delivery of HS100 spheroids significantly decreased the levels of pro-inflammatory cytokines such as IL-2, IL-3, IL-6, IFN-γ and TNF-α in the serum. Additionally, flow cytometry data from retrieved lymph nodes showed that delivery of HS100 spheroids significantly reduces the generation of both effector memory T cells and central memory cells (272).

In summary, both hypo-immune islets and encapsulated islets are being studied to improve islet transplantation outcomes and achieve normoglycemia in type 1 diabetes patients. The use of hypoimmune cells offers advantages over encapsulation by eliminating diffusion barriers between the cells and the native environment, allowing for faster glucose sensing and insulin release by the transplanted cells. Without encapsulation, nutrients, oxygen, and waste can freely exchange with the bloodstream, leading to quicker metabolic responses. Additionally, hypoimmune cells can be infused into the hepatic portal vein, eliminating the need for surgical or invasive procedures. However, gene editing techniques pose potential risks, including tumorigenicity, which remains a safety concern. In contrast, the use of encapsulation devices creates a barrier that slows nutrient and oxygen exchange, requiring additional technological advancements to maintain cell viability, such as oxygenation strategies or vascularization enhancements, as well as long-term evasion of foreign body response. Despite this limitation, encapsulation devices provide other advantages such as higher localization of the transplanted cells, potentially reducing the loss of cells associated with cell infusions into the hepatic portal vein (200).

These benefits motivated significant preclinical work to incorporate immunomodulatory agents within biomaterials for prolonging graft survival while minimizing the need for systemic immunosuppression. Although localized delivery of immunomodulators, within the encapsulated device or co-delivered along with naked islets, is promising in extending the survival of transplanted islets *in vivo*, they often need complementary systemic immunosuppressors to be used in tandem for long-term results. This can further complicate the treatment protocol. Moreover, most of these delivery systems require the replenishment of immunomodulatory agents. These limitations can hinder the clinical translatability of these strategies, adding to the complexity and cost of manufacturing.

## 5 Conclusion and outlook

Recent progress in understanding the immune mechanisms behind the pathogenesis of type 1 diabetes has accelerated the development of preventative therapies. These efforts led to FDA approval of the first disease-modifying drug and increased clinical interest in immunotherapies for the prevention. One notable example is that immune blockade therapies have been shown to be efficacious in clinical trials. However, systemic administration and broad immune targeting often lead to severe adverse effects, limiting their use as early-stage interventions, when their use could be most effective. Alternatively, antigen-specific and cell-based immunotherapies are explored as more targeted options, but they have shown limited efficacy. Antigen-specific strategies also have demonstrated variability in patient response and delivery limitations, while cell-based therapies have been shown to exhibit off-target effects and face challenges in isolation and expansion. These advances show the promise and complexity of designing efficacious and targeted interventions for disease prevention.

In parallel, β-cell replacement therapies are being developed to restore endogenous insulin production for patients at the late stage of the disease. Clinical milestones such as FDA approval of allogeneic islet transplantation and ongoing phase 3 clinical trials with stem cell-derived islets have demonstrated the potential of these solutions. Yet, their continued dependence on systemic immunosuppression poses safety and durability limits their broad application. To address this, several immunomodulatory approaches are being explored to create an immune-privileged environment around transplanted islets. However, these systems either lack long-term efficacy, still require systemic immunosuppression, or frequently necessitate the replenishment of immunomodulatory agents. All these factors exacerbate manufacturing complexity and cost. Additionally, the foreign body response to biomaterial-based platforms, limited vascularization, and inadequate oxygen supply at some implantation sites compromise islet viability and functionality. Moreover, clinical trials have also revealed a significantly lower islet survival rate than preclinical models, reflecting a gap between experimental findings and real-world outcomes.

In summary, while substantial progress has been made in immunomodulation strategies and β-cell replacement, there are still significant obstacles. Advancing these therapies requires addressing issues such as immune specificity, long-term efficacy, and clinical scalability, which will be critical for translating these promising approaches into effective and widely accessible treatments for T1D.
